# Evolution of competence and DNA uptake specificity in the Pasteurellaceae

**DOI:** 10.1186/1471-2148-6-82

**Published:** 2006-10-12

**Authors:** Rosemary J Redfield, Wendy A Findlay, Janine Bossé, J Simon Kroll, Andrew DS Cameron, John HE Nash

**Affiliations:** 1 Dept. of Zoology, University of British Columbia, Vancouver BC Canada; 2 Institute for Biological Sciences, National Research Council of Canada, Ottawa ON Canada; 3 Dept. of Paediatrics, Faculty of Medicine, Imperial College London, London W2 1PG UK; 4 Dept. of Microbiology and Immunology, University of British Columbia, Vancouver BC Canada

## Abstract

**Background:**

Many bacteria can take up DNA, but the evolutionary history and function of natural competence and transformation remain obscure. The sporadic distribution of competence suggests it is frequently lost and/or gained, but this has not been examined in an explicitly phylogenetic context. Additional insight may come from the sequence specificity of uptake by species such as *Haemophilus influenzae*, where a 9 bp uptake signal sequence (USS) repeat is both highly overrepresented in the genome and needed for efficient DNA uptake. We used the distribution of competence genes and DNA uptake specificity in *H. influenzae*'s family, the *Pasteurellaceae*, to examine the ancestry of competence.

**Results:**

A phylogeny of the *Pasteurellaceae *based on 12 protein coding genes from species with sequenced genomes shows two strongly supported subclades: the *Hin *subclade (*H. influenzae, Actinobacillus actinomycetemcomitans*, *Pasteurella multocida, Mannheimia succiniciproducens*, and *H. somnus*), and the *Apl *subclade (*A. pleuropneumoniae*, *M. haemolytica*, and *H. ducreyi*). All species contained homologues of all known *H. influenzae *competence genes, consistent with an ancestral origin of competence. Competence gene defects were identified in three species (*H. somnus, H. ducreyi *and *M. haemolytica*); each appeared to be of recent origin.

The assumption that USS arise by mutation rather than copying was first confirmed using alignments of *H. influenzae *proteins with distant homologues. Abundant USS-like repeats were found in all eight Pasteurellacean genomes; the repeat consensuses of species in the *Hin *subclade were identical to that of *H. influenzae *(AAGTGCGGT), whereas members of the *Apl *subclade shared the consensus ACAAGCGGT. All species' USSs had the strong consensus and flanking AT-rich repeats of *H. influenzae *USSs. DNA uptake and competition experiments demonstrated that the *Apl*-type repeat is a true USS distinct from the *Hin*-type USS: *A. pleuropneumoniae *preferentially takes up DNA fragments containing the *Apl*-type USS over both *H. influenzae *and unrelated DNAs, and *H. influenzae *prefers its own USS over the *Apl *type.

**Conclusion:**

Competence and DNA uptake specificity are ancestral properties of the *Pasteurellaceae*, with divergent USSs and uptake specificity distinguishing only the two major subclades. The conservation of most competence genes over the ~350 million year history of the family suggests that lineages that lose competence may be evolutionary dead ends.

## Background

Many bacteria are able to take up DNA from the environment [1]. DNA provides these naturally competent cells with nutrients (nucleotides, N and P), while recombination of incoming DNA with the cell's genome can also provide new genetic information. However, many aspects of the evolution of competence remain unclear.

Competence is widely distributed among bacteria, and some of the genes required for DNA uptake are shared between even distant relatives, suggesting an ancient common origin for competence. For example, the Gram positive bacteria *Bacillus subtilis *and *Streptococcus pneumoniae *and the Gram negative *Neisseria gonorrhoeae *and *Haemophilus influenzae *all require homologues of type four pilus proteins and of the ComEC/Rec2 membrane channel [1]. However, the regulatory processes controlling expression of these competence genes are very different in the different organisms [2]. Furthermore the distribution of natural competence is surprisingly sporadic; most naturally competent bacteria have many relatives, including other strains of the same species, that cannot be transformed under laboratory conditions (for examples see [3-6]). Two explanations seem equally plausible. First, competence might be ancestral to most major lineages but frequently lost (and possibly regained, under different regulation). Alternatively, competence might be frequently gained in independent lineages, *e.g*. if the genetic requirements for DNA uptake are simple and readily met by laterally transferred genes or by mutation of genes with related functions such as those associated with type IV pili.

The uptake specificity of some naturally competent bacteria can also guide inferences about the evolution of competence. Although many naturally competent bacteria will take up DNA fragments from any source with equal efficiency, members of some Gram-negative families take up DNA fragments from their own species much more efficiently than unrelated DNA. In the *Pasteurellaceae *and *Neisseriaceae *the molecular basis of this specificity is preferential binding of the uptake machinery to short DNA sequences present in thousands of copies in each species' genome. Such sequences are referred to as uptake signal sequences (USSs) in the *Pasteurellaceae *and DNA uptake sequences (DUSs) in the *Neisseriaceae*; they are not known in other naturally transformable bacteria [7,8].

The best-characterized uptake sequences are those of *Haemophilus influenzae *and *Neisseria meningitidis *and *N. gonorrhoeae*. The preferred sequences themselves appear to have little in common: the core *H. influenzae *USS is 5'-AAGTGCGGT (5'-ACCGCACTT in the reverse orientation), with two AT-rich motifs on the 3' side of the standard orientation [9], whereas the *Neisseria *DUS is GCCGTCTGAA with no flanking motifs [10]. However, similarities in genomic frequencies and distributions suggest that they have arisen by similar processes. Both USSs and DUSs are present in their respective genomes at frequencies close to one copy per kb and both show no significant orientation bias. Both types are distributed somewhat more regularly around their genomes than expected for randomly located repeats, but both have some copies occurring in closely spaced oppositely oriented pairs [7]. Both USSs and DUSs are preferentially found in non-coding DNA sequences, but both have many copies in coding sequences. Both types are also overrepresented in the genomes of at least some other members of their genus or family [4,7-9,11-13].

One puzzling attribute shared by USSs and DUSs is an unusually strong consensus, with each genome containing many more copies that perfectly match its consensus core sequence than singly mismatched copies. This pattern is typical of young transposons and other genetic elements that multiply by copying, but very different from the more relaxed consensus typical of sequences that function as binding sites for regulatory proteins, which arise by point mutation of pre-existing sequences. Because USSs are thought to function by binding to DNA-receptor proteins at the cell surface [14,15], their very strong consensus is anomalous. The explanation might be that the DNA uptake machinery at the cell surface binds DNA with much higher specificity than do intracellular DNA-binding proteins. However the possibility that USSs arise by a copying process has not been excluded.

Previous analysis has found that copies of the *H. influenzae *USS are abundant in the genomes of several other members of the *Pasteurellaceae *(*Actinobacillus actinomycetemcomitans, Pasteurella multocida*, and *H. somnus*), and comparison of homologous genes in *H. influenzae *and *P. multocida *has shown that individual USSs can be stable over hundreds of millions of years [13]. However, preliminary examinations of the sequenced genomes of the Pasteurellaceans *Mannheimia haemolytica *(by Sarah Highlander) and *Actinobacillus pleuropneumoniae *(by ourselves) found that the *H. influenzae *USS was much less abundant than a related sequence that differs at several positions, suggesting that these genomes might contain a variant USS.

Insight into the evolution of competence will depend on an improved understanding of Pasteurellaceaen phylogeny. Almost all *Pasteurellaceae *(gamma-proteobacteria) are commensals and/or pathogens of the mucosal surfaces of vertebrates, primarily birds and mammals, and several are important human pathogens. Although phylogenetic analysis based on 16S rRNA sequences has confirmed that the family is monophyletic, the relationships of its members remain poorly resolved. Two recently published phylogenies used small-subunit rRNA sequences from 83 Pasteurellacean taxa [16] and partial sequences of the housekeeping genes *atpD, infB *and *rpoB *from 28–36 strains [17], but the resolution was unsatisfactory, with many low bootstrap values and unresolved nodes.

Here we use the concatenated sequences of 12 proteins to construct a well-resolved phylogenetic tree for the species of Pasteurellaceae with genome sequences available. This tree then serves as a framework against which we characterize the long-term evolution of competence genes and of DNA uptake specificity.

## Results and Discussion

### A robust Pasteurellacean phylogeny

**Figure 1:**
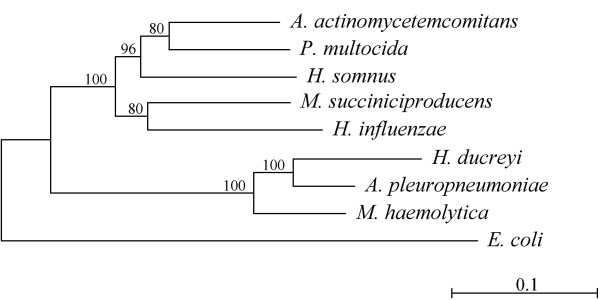
**Phylogeny of 8 Pasteurellacean species. **Phylogenetic analysis was based on amino acid sequence of 12 protein-coding genes (listed in **Methods**) with homologues in all 8 Pasteurellacean genomes and in *E. coli*. The scale bar is 0.1 substitutions per site.

The amino acid sequences of 12 well-conserved genes were identified from the available published and draft sequences of Pasteurellacean genomes and used to infer the consensus phylogeny shown in Figure 1. Homologous *E. coli *genes were used as the outgroup. The chosen genes did not contain the *H. influenzae *USS, were distributed around the *H. influenzae *genome, and had strong homologues in the other genomes. Intracellular proteins were chosen to preclude the diversifying selection that can bias evolution of proteins exposed on the cell surface, and genes with base compositions typical of their species' genomes were used to preclude recent horizontal transfer.

The resulting phylogenetic tree (Fig. 1) identified two primary subclades within the *Pasteurellaceae*. These are referred to below as the *Hin *subclade and the *Apl *subclade. The statistical support for these subclades is robust, with bootstrap values of 100%. Independent phylogenies done for each protein separately all grouped *A. pleuropneumoniae, M. haemolytica *and *H. ducreyi *together as a distinct subclade from the five other species. The 100% bootstrap values place *A. pleuropneumoniae *and *H. ducreyi *as sister species, but the branching order in the *Hin *subclade remains uncertain.

This phylogeny is restricted to the eight species with sequenced genomes, but it is the first Pasteurellacean tree to have strong statistical support. It differs in many respects from both the 16S rRNA and protein phylogenies previously published for the Pasteurellaceae. However in our view these discrepancies are the consequence of most branches of those earlier phylogenies having very poor bootstrap support, making them intrinsically unreliable, and so should not be a cause for concern. The *Apl *subclade it predicts was also supported by the protein tree of Christensen *et al*. [17]. Although the *Apl *subclade is not seen in Christensen *et al*.'s small-subunit rRNA tree, the resolution of that region of their tree is poor (best bootstraps are 69% and 75%) [16]. The topology of an earlier tree based on small subunit rRNA sequences agrees with ours, although none of the relevant bootstrap values in that tree are significant [18]. The new tree also confirms what the previous more-detailed but less-well-supported trees had predicted – that within the *Pasteurellaceae *true evolutionary relatedness is not well correlated with many of the features previously used to assign isolates to genus [17,19,20].

The genus assignment of *M. succiniciproducens *provides an example. This species, isolated from bovine rumen, was assigned to *Mannheimia *based on a simple small-subunit rRNA tree with no bootstrap analysis [21]. Our phylogenetic analysis instead places the two *Mannheimia *species in separate subclades. The 80% bootstrap score supporting *M*. *succiniciproducens*'s placement as the sister group to *H. influenzae *in Fig. 1 is too low to rule out a closer affinity with *P. multocida*. Hong *et al*. compared the *M*. *succiniciproducens *genome sequence to those of both *P. multocida *and *H. influenzae*; more genes are shared with the former, but the amino acid identities are higher with the latter [22,23]. In any case it is striking to find a rumen bacterium as such a close relative of bacteria otherwise restricted to respiratory mucosa.

### Competence genes in Pasteurellacean genomes

Natural transformation has been demonstrated experimentally in only three of the eight sequenced species (*H. influenzae, A. actinomycetemcomitans *and *A. pleuropneumoniae *[12,24,25]). Only two other species within the *Pasteurellaceae *have also been shown to be naturally competent (*Haemophilus parasuis *[26] and *Haemophilus parainfluenzae *[4,27]). A number of other species have resisted multiple attempts at transformation in the laboratory, but their nontransformability could be misleading, as cellular processes important in the natural environment may not be induced under laboratory culture conditions.

VanWagoner *et al*. identified homologues of several *H. influenzae *competence genes (HI0366, HI0938 and HI0939) in most sequenced Pasteurellacean genomes [28]. As we have recently identified the complete competence regulon of *H. influenzae*, we examined the genomes of all of the sequenced *Pasteurellaceae *for homologues of all of these genes [29]. Table 1 shows that all of the genomes contain recognizable homologues of all of the genes known to be required for competence in *H. influenzae*, as well as homologues of most other genes consistently occurring in the same operons.

**Table 1:** Competence genes in Pasteurellacean genomes

Gene	HI#	Species							
		*Hin*	*Aac*	*Pmu*	*Hso*	*Msu*	*Apl*	*Mha*	*Hdu*

*comA*	0439	+	+	+	+	+	+	+	def
*comB*	0438	+	+	+	+	+	+	+	def
*comC*	0437	+	+	+	+	+	+	+	+
*comD*	0436	+	+	+	def	+	+	+	+
*comE*	0435	+	+	+	def	+	+	+	+
*comF*	0434	+	+	+	+	+	+	+	+
*comE1*	1008	+	+	+	+	+	+	+	+
*comM*	1117	+	+	+	+	def	+	+	def
*dprA*	0985	+	+	+	+	+	+	+	+
*pilA*	0299	+	+	+	+	+	+	+	+
*pilB*	0298	+	+	+	+	+	+	def	+
*pilC*	0297	+	+	+	+	+	+	+	+
*pilD*	0296	+	+	+	+	+	+	+	+
*rec2*	0061	+	+	+	+	+	+	+	+
*sxy*	0601	+	+	+	+	+	+	+	+
	0366	+	+	+	+	+	+	+	+
	0938	+	+	+	+	+	+	+	+
	0939	+	+	+	+	+	+	+	+

+ Gene present

def Gene present but defective due to mutation

However not all of the genes in this ancestor's sequenced descendants appear to be functional. The *H. ducreyi comA, comB *and *comM *genes are interrupted by an internal stop codon (*comA*) and frameshifts (*comB *and *comM*). A deletion in the *H. somnus *genome fuses the 5' portion of *comD *to the 3' 67% of *comE*, which also contains a frameshift. A 17 kb insertion disrupts the *comM *gene of *M. succiniciproducens*, and most of *pilB *in *M. haemolytica *has been deleted. In some cases, examination of genome sequences from different isolates revealed discrepancies; these may result from strain-specific variation or from the preliminary nature of some of the sequences used. Only the sequenced genomes of *H influenzae, A. actinomycetemcomitans, P. multocida *and *A. pleuropneumoniae *retain fully intact sets of competence genes.

What inferences can be drawn about the evolution of competence? First, the most parsimonious explanation for the presence of all competence genes in all genomes is that the ancestral Pasteurellacean had functional copies of all these genes and was naturally competent. When and where did this ancestor live? Although dating bacterial divergences is highly problematic, the most recent common ancestor of *H. influenzae *and *P. multocida*, and thus of the *Hin*-subclade in Fig. 1, has been estimated to have lived about 270 million years ago (mya), and last common ancestor of the entire family must be older still [30,31]. Thus the origin of the *Pasteurellaceae *is likely to have long predated the origin of mammals (*c*.195 mya) and may be contemporaneous with the origin of tetrapods about 360 mya. If so, it is possible that these bacteria moved into the respiratory tract and used the abundant DNA found there [32] almost as soon as the first respiratory tracts evolved.

What then explains the sporadic distribution of competence in its descendants? Three of the five genomes from 'non-transformable' species we analyzed carry obvious genetic defects that would prevent DNA uptake. (Loss of *comM *in *M. haemolytica *would only prevent transformation.) Each defect is unique and so must have arisen since the most recent divergence in its lineage. Furthermore, the substitution rate indicated by the scale bar on Fig. 1 allows estimation of the minimum number of chain-terminating and frameshift mutations expected to have accumulated since loss of a competence gene removed selection on other competence-specific genes. The scarcity of such mutations in each of these strains (0, 1 or 2) suggests that competence was lost quite recently. This is consistent also with the high densities and strong consensuses of the USS in all genomes except *H. ducreyi*. Frequent recent losses of competence would also explain the reported variation in competence within populations [3-6].

### Uptake signal sequences (USS) are not insertions

One goal of this work was to use USS distribution to make inferences about the evolution of DNA uptake specificity. However, the anomalously strong consensuses of *H. influenzae *USSs (and other USSs) raised the concern that they might have been produced by insertion of a replicating element rather than by point mutations in pre-existing sequences. Fortunately the mode of USS origin makes a simple prediction about the positions of gaps in sequence alignments. If individual *H. influenzae *USSs have arisen by insertion, gaps should be seen when the segments containing these USS are aligned with homologous sequences from genomes that diverged before USS arose. We used this prediction to test whether the many *H. influenzae *USSs in protein coding sequences arose in an ancestral Pasteurellacean by insertion or by accumulation of point mutations in the ancestral genes.

**Figure 2:**
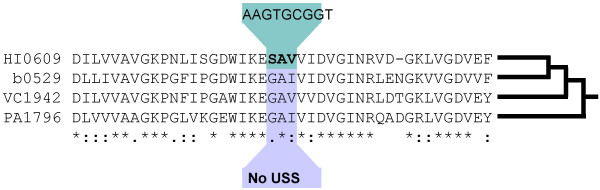
**Example gap analysis of a USS-homologous peptide. **A USS-encoded peptide and flanking region within the *H. influenzae folD *gene (HI0609; amino acids 203–245), aligned with *folD *proteins from: *E.coli *(b0529; amino acids 203–246), *Vibrio cholerae *(VC1942; amino acids 228–271), and *Pseudomonas aeruginosa *(PA1796; amino acids 203–246). The sketch at the right shows the phylogenetic relationships of these taxa [57].

Because of the evolutionary distance between *H. influenzae *and species with no USS, the alignments were done between predicted amino acid sequences rather than nucleotide sequences. Segments of well-conserved *H. influenzae *proteins, centred on USS-encoded amino acids, were aligned with homologous protein segments from *Escherichia coli, Vibrio cholerae *and *Pseudomonas aeruginosa*, whose genomes do not contain USS-like repeats. A typical alignment is shown in Fig 2, along with a sketch of the evolutionary relationships of these bacteria.

Of the 956 *H. influenzae *USS in protein-coding regions (65% of all USS), 158 were in protein segments that could be aligned with = 50% identity to homologues from all three other species. Only 24% (115/474) of the individual *E. coli, V. cholerae *and *P. aeruginosa *proteins alignments contained gaps, and most gaps were outside of the segment encoded by the USS core. Similar results were seen in alignments between homologues from outside the gamma-proteobacteria and the subset of *H. influenzae *protein sequences sufficiently well conserved to align well (results not shown). The homology between amino acids that are USS-encoded in *H. influenzae *and amino acids in distant relatives confirms that USSs have arisen by nucleotide substitutions in pre-existing sequences and not by insertions of a replicative element.

### All Pasteurellacean genomes contain USS-like repeats

The next step was to characterize the phylogenetic distribution of USS. Bakkali *et al*. found that the only overrepresented short repeats in the *P. multocida *genome are variants of the 9 bp *H. influenzae *USS core [13]. They also found the *H. influenzae *USS core to be highly overrepresented in the *H. somnus *and *A. actinomycetemcomitans *genomes but did not survey other repeats. To avoid the bias of searching for a specific USS sequence, we extended this analysis by counting all 6–12 bp repeats in all eight genomes (Table 2) and calculating the number of each repeat expected in a random-sequence genome of the same size and base composition.

**Table 2:** Most common 8-, 9- and 10-mers in Pasteurellacean genome sequences

Genome^a^	%G+C	Size (Mb)	Most common 10-mer^b ^(number, fold over-rep)	Most common 9-mer^b ^(number, fold over-rep)	Most common 8-mer^b ^(number, fold over-rep)
*Hin*	38.1	1.8	AAAGTGCGGT (1115, 429X)	AAGTGCGGT (1471, 175X)	AAGTGCGG (1687, 63X)
*Aac*	44.4	2.1	AAAGTGCGGT (1422, 384X)	AAGTGCGGT (1760, 132X)	AAGTGCGG (1863, 39X)
*Pmu*	40.4	2.3	AAAGTGCGGT (700, 200X)	AAGTGCGGT (927, 79X)	AAGTGCGG (1013, 26X)
*Hso*	37	2.1	AAAGTGCGGT (776, 273X)	AAGTGCGGT (1216, 135X)	AAGTGCGG (1446, 51X)
*Msu*	42.5	2.3	AAAGTGCGGT (1297, 333X)	AAGTGCGGT (1485, 111X)	AAGTGCGG (1622, 46X)
*Apl*	41.4	2.2	ACAAGCGGTC (429, 187X)	ACAAGCGGT (742, 68X)	CAAGCGGT (1361, 36X)
*Mha*	41	2.7	ACAAGCGGTC (506, 181X)	ACAAGCGGT (973, 70X)	CAAGCGGT (1636, 48X)
*Hdu*^c^	38.2	1.76	TTTTGCAAAA (106, 9.6X) AATAAGCGGT^c ^(95, 21X) AACAAGCGGT^c ^(85, 31X)	AATAAAAAA (251, 2.8X) ACAAGCGGT^c ^(199, 23X)	AAAAATAA (680, 2.3X) CAAGCGGT^c ^(464, 16X)

^a ^*Hin: H. influenzae; Aac: A.s actinomycetemcomitans; Pmu: P. multocida; Hso: H. somnus; Msu: M. succiniciproducens; Apl: A. pleuropneumoniae; Mha: M. haemolytica; Hdu: H. ducreyi*.

^b ^The first number in parentheses is the combined number of copies of the sequence and its reverse complement. The second number in parentheses is the fold over-representation of each repeat compared to a random-sequence genome of the same size and base composition.

^c ^The additional entries for *H. ducreyi *are the copy numbers of the most frequent USS-like repeats.

Table 2 shows that all genomes had highly overrepresented repeats related to the *H. influenzae *USS. The most common 9-mer repeats in the genomes of *A. actinomycetemcomitans, P. multocida, M. succiniciproducens *and *H. somnus *are the *H. influenzae *USS core AAGTGCGGT and its reverse complement. All of the ten most abundant 8-mer, 9-mer and 10-mer repeats in these genomes also contain or closely overlap this 9-mer. We will refer to this as the *Hin*-type USS. However the most frequent 9-mer repeats in the genomes of *A. pleuropneumoniae *and *M. haemolytica *differed from the *Hin*-type USS at the second, third and fourth positions (ACAAGCGGT rather than AAGTGCGGT); we will refer to this as the *Apl*-type USS. The most abundant repeats in the *H. ducreyi *genome were not recognizable USSs but simple palindromes and strings of As and Ts, so Table 2 also gives the frequencies of the most common USS-like 8, 9 and 10-mers for this genome. These resembled the *Apl*-type USS but their copy numbers were substantially lower than in the other genomes. (Although the 10-mer AATAAGCGGT was the most common USS-like 10-mer repeat, ATAAGCGGT and TAAGCGGT were not among the 50 most frequent 9-mers and 8-mers.)

**Figure 3:**
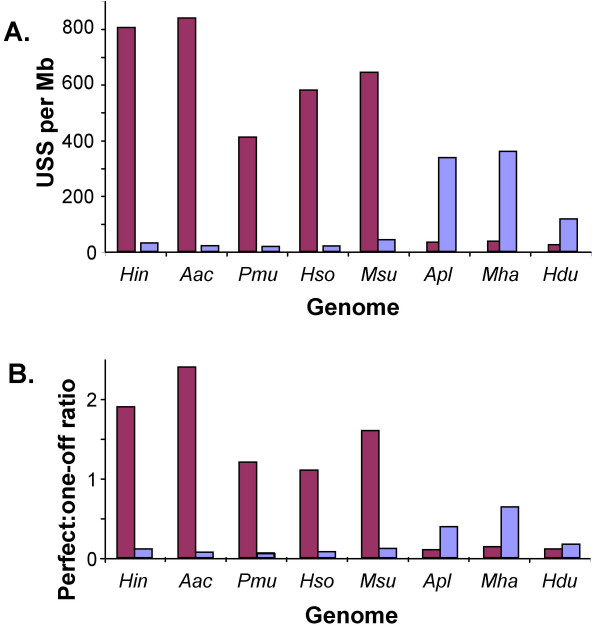
**USS frequencies in sequenced Pasteurellacean genomes. **Red: *Hin*-type USSs (AAGTGCGGT); blue: *Apl*-type USSs (ACAAGCGGT). **A. **Frequencies of 9 bp core USSs of each type per Mb of genome. **B. **Ratios of perfect to singly mismatched 9 bp USS cores.

Each genome was specifically checked for repeats of the other USS type. The frequencies of both types of 9 bp USSs per Mb sequence in all eight genomes are shown in Fig. 3A. Only 4 copies per Mb would be expected in random-sequence genomes of the same base compositions. Although the minority USS type (*e.g. Hin*-type USS in *A. pleuropneumoniae*) is several-fold overrepresented in each genome, it is not significantly more frequent than other 9-mers sharing the global consensus ANNNGCGGT. Thus each genome appears to have a predominant subclade-specific USS type.

Fig. 3B shows, for each Pasteurellacean genome, the ratio of repeats perfectly matching each USS type to repeats with single mismatches to that type. Genomes with *Hin*-type USSs resemble *H. influenzae *in having more perfect than singly mismatched copies, despite the 27-fold greater number of possible sequences. The discrepancy is also seen for genomes with *Apl*-type USSs; with the exception of *H. ducreyi*, the ratio is substantially higher for the subclade-specific USS type than for the other type. The consistency of the pattern suggests that USS accumulation is shaped by similar forces in the different genomes.

### Detailed comparisons of USSs

**Figure 4:**
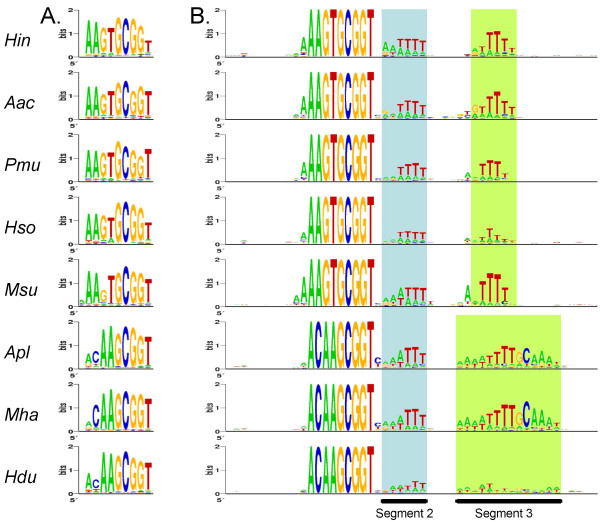
**WebLogos for USSs and surrounding sequence in 8 genomes. A. **Logos based on 9 bp segments with perfect or one-off matches to the 9 bp USS. **B. **Logos based on 50 bp segments with perfect matches to the 9 bp USS.

**Figure 5:**
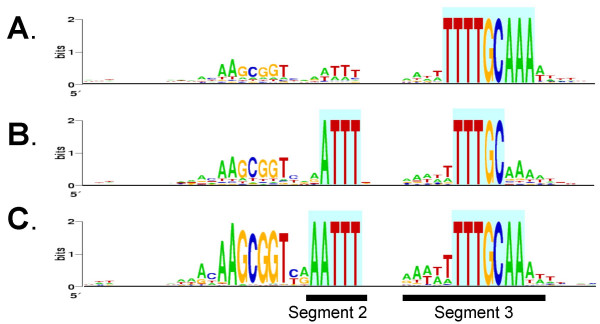
**WebLogos for 50 bp segments of the *A. pleuropneumoniae *genome. **The highlighting indicates the positions used to choose sequences for analysis.**A. **Logos for segments containing the motif TTTTGCAAA. **B. **Logos for segments containing the motif ATTTNNNNNNNNNTTTGC. **C. **Logos for segments containing the motif AATTTNNNNNNNNNTTTGCAA.

As USSs are thought to function by binding to DNA receptors on the cell surface, bases at different positions in the USS core would be expected to show consensus strengths reflecting their differing contributions to this DNA-protein binding. Sequence logos were used to visualize the representation of each base at each position of the USS (Figs. 4 and 5) [33]. In these logos the relative heights of the A, G, C and T in each stack shows the frequencies of the bases at that position, and the overall height of each stack of letters reflects the strength of the consensus at that position (the information content). The height of the stack is especially sensitive to minor changes in the frequency of a very frequent base (*e.g*. if the frequency of the most common base falls from 1.0 to 0.9 the height falls from 2.0 to 1.6).

Fig. 4A shows the differences in consensus strengths at the USS core positions. In all genomes with *Hin*-type USSs most core positions appear to have roughly equal consensus strengths, suggesting that all make similar contributions to binding specificity. The exceptions are the final T, which has a weaker consensus in *H. influenzae *and *H. somnus*, and the first G in *M. succiniproducens*. For genomes with the *Apl*-type USS the first two positions of USS cores show a weaker consensus than the other positions, suggesting that they may make a lesser contribution to the specificity of DNA binding and uptake. Confirmation of these predictions must await identification and characterization of the proteins or other molecules that interact with the USS at the cell surface.

The *H. influenzae *and *A. actinomycetemcomitans *USSs have been shown to also share conserved motifs (segments 2 and 3) on the 3' side of the USS core [9,12]. The importance of segment 2 was experimentally demonstrated by Danner and coworkers, who showed that USS-containing DNA fragments ethylated at bases in this region were not taken up by competent *H. influenzae *cells [15]. The functions of these positions in DNA uptake are not known; they may be additional sites of contact with the DNA receptor, or they may be involved in DNA bending or kinking during uptake.

Fig. 4B shows the consensuses of positions flanking the core USSs in each of the eight genomes. As viewed in the standard orientation, all USS consensuses have an AT-rich motif just 3' of the core (segment 2; positions 22–27 as numbered in Fig. 4B), with the first bases usually 'A's and the final 3 or 4 bases 'T's. A second AT-rich motif is seen further downstream (segment 3). In the *Hin*-type USS this 6nt-motif segment consists primarily of Ts, and is centred 12 positions to the right of segment 2. In *A. pleuropneumoniae *and *M. haemolytica*, segment 3 extends slightly farther to the left and substantially farther to the right, and has the more complex consensus AAAATTTTGCAAAT. Although the *H. ducreyi *USS consensus in segments 2 and 3 resembles the *Apl*-type motifs, it is much weaker. Together with the lower frequency of USS in its genome, and the presence of inactivating mutations in three of its competence genes, this suggests a relatively ancient loss of ability to take up DNA.

The consensuses in segments 2 and 3 of the *A. pleuropneumoniae *and *M. haemolytica *USSs were particularly strong and extensive. To compare their strengths to that of the core USS we repeated the above analysis in reverse. We chose the nine bases making the strongest contribution to segment 2 and segment 3 (ATTTNNNNNNNNNTTTGC) or to segment 3 alone (TTTTGCAAA) and identified and aligned all *A. pleuropneumoniae *genomic segments containing them (560 and 454 segments respectively). The resulting logos (Fig. 5A and 5B) show that many of the segments bearing these motifs also contained all but the first two bases of the *Apl*-type USS core. The weak correlation of the first two positions of the core with the flanking segments may mean that these positions play a lesser role in USS function than the rest of the core, with its larger segment 3 making a greater contribution to the binding specificity. A logo using only the 164 sequences with 12 matches to segments 2 and 3 was even more effective, recovering the full core consensus (Fig. 5C). Taken together, these analyses suggest that, at least in *A. pleuropneumoniae*, the motifs in segments 2 and 3 may be as important for DNA uptake as the USS core.

### USS in H. parasuis

A recent paper reported that *H. parasuis *has the core USS GAGTTCGGT, which differs from both the *Hin *and *Apl *types[26]. However this conclusion was based on analysis of a single putative USS in a cloned 413 bp fragment. We have examined all the available *H. parasuis *sequences (86,701nt, mainly in ORFs) and find, in addition to the one copy of this repeat described by Bigas *et al*., four copies of the *Hin*-type USS, fourteen copies of the *Apl*-type USS, and seventeen copies of sequences differing at single positions from the *Apl*-type USS. This suggests that *H. parasuis *has an *Apl*-type USS, which would be consistent both with previous phylogenetic analysis placing it in a strongly supported subclade with *M. haemolytica *and *A. pleuropneumoniae *[17,34] and with the ability of *A. pleuropneumoniae *DNA to efficiently transform *H. parasuis *[35].

### H. influenzae and A. pleuropneumoniae recognize subclade-specific USSs

*A. actinomycetemcomitans *(*Hin*-type USS) has already been shown to preferentially take up its own and *H. influenzae *DNAs [12], but for most of the other species the role of the putative USS in DNA uptake could not be directly tested because no competent isolate has been identified. However *A. pleuropneumoniae *strain HS143 (serotype 15) has recently been shown to be much more competent than other strains (J. Bossé, manuscript in preparation), allowing us to test its uptake specificity by three different experiments. Each confirmed that competent *A. pleuropneumoniae *cells preferentially take up DNA fragments containing the *Apl*-type USS.

**Figure 6:**
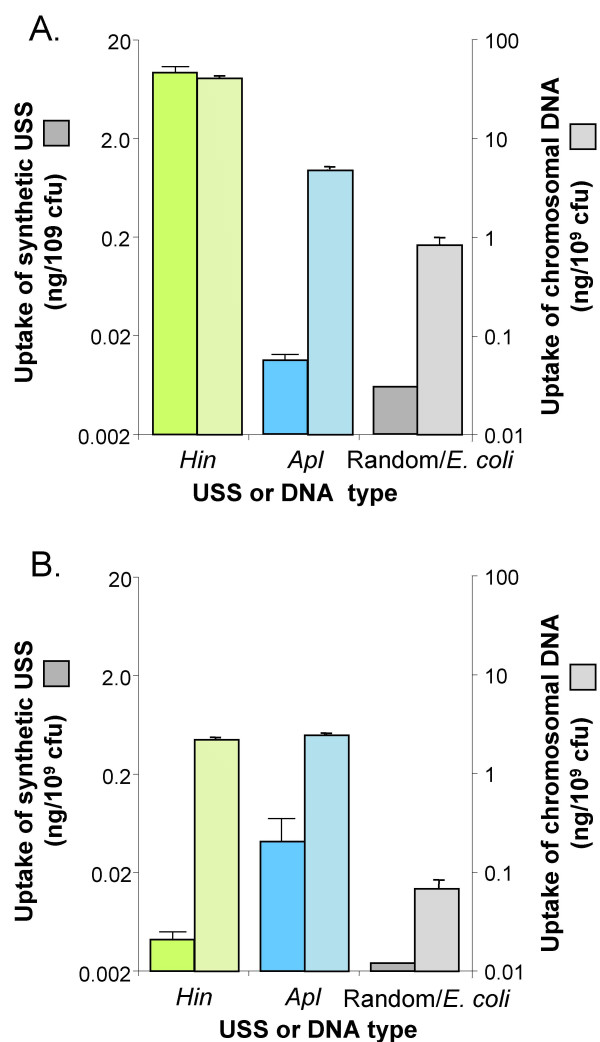
**Uptake of synthetic USSs or chromosomal DNAs. **The solid bars show uptake of 220 bp PCR fragments containing synthetic USS with the consensus sequences of *H. influenzae *(*Hin*), *A. pleuropneumoniae *(*Apl*) or randomized *H. influenzae *USS types. The dashed bars show uptake of chromosomal DNAs from *H. influenzae *(*Hin*), *A. pleuropneumoniae *(*Apl*) or *E. coli*. Error bars show the standard deviations of 3 replicate experiments, except for *A. pleuropnemoniae *in **A**, which is from 4 replicate experiments. **A. **Uptake by *H. influenzae*. **B. **Uptake by *A. pleuropneumoniae*.

The solid bars in Figure 6A and 6B show measurements of uptake by competent *H. influenzae *and *A. pleuropneumoniae *cells of radiolabelled 220 bp DNA fragments containing synthetic *H. influenzae *and *A. pleuropneumoniae *USSs. These USSs were designed to contain the most common base at each position of the extended USSs described above; a control fragment contained a randomized version of the *H. influenzae *USS sequence. As expected, *H. influenzae *took up about 1500-fold more DNA containing its USS than control DNA (Fig. 6A; note the log scale). The function of the *Apl*-type putative USS was confirmed; *A. pleuropneumoniae *took up about 17-fold more DNA with its USS than control DNA (Fig. 6B). Each species also took up substantially less DNA containing the heterologous USS type than its own type (only about twice as much as control DNA), confirming that the DNA uptake machinery discriminates between the two types.

Uptake of chromosomal DNA may provide a more biologically relevant measure of specificity. The dashed bars in Fig. 6A and 6B show uptake of radiolabelled chromosomal DNAs by competent H. *influenzae *and *A. pleuropneumoniae *cells. In this assay *H. influenzae *took up 50-fold more *H. influenzae *DNA than the control *E. coli *DNA (Fig. 6A), and *A. pleuropneumoniae *took up about 37-fold more *A. pleuropneumoniae *DNA than *E. coli *DNA (Fig. 6B). In both chromosomal and synthetic-USS uptake experiments *A. pleuropneumoniae *took up substantially less DNA than did *H. influenzae*, consistent with its lower transformation frequency.

Consideration of the relative densities of the two USS types in the three genomes (*H. influenzae: A. pleuropneumoniae: E. coli*; 198:8:1 (*Hin*-type USSs) and 4.4:48:1 (*Apl*-type USSs)) allows clarification of the degree to which each species takes up DNA of the other type. First, the high density of *Hin*-type USS in the *H. influenzae *genome means that most chromosomal DNA fragments (≥ 50 kb long) would have contained at least several USS, reducing the contribution of each USS to uptake. Second, the presence of heterologous USS in the two Pasteurellacean genomes can explain *H. influenzae*'s 6-fold higher uptake of *A. pleuropneumoniae *DNA than *E. coli *DNA, but is likely insufficient to explain *A. pleuropneumoniae*'s 32-fold higher uptake of *H. influenzae *DNA than *E. coli *DNA. These results suggest that the *A. pleuropneumoniae *uptake machinery does indeed weakly recognize the *Hin*-type USS, and do not preclude a similar overlap in specificity by the *H. influenzae *uptake machinery.

**Figure 7:**
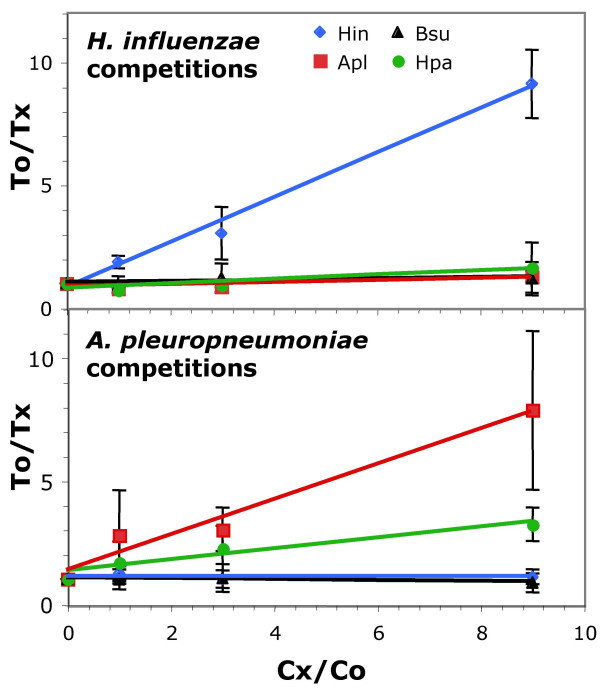
**Double-reciprocal plots of uptake competition assays. **Double-reciprocal plots of uptake competition assays. Cx/Co: ratio of competing DNA to genetically marked self DNA. To/Tx: ratio of number of transformants in the presence and absence of competing DNA Competing DNAs: blue diamonds, *H. influenzae*; red squares, *A. pleuropneumoniae*; black triangles, *B. subtilis*; green circles, *H. parasuis*. **A. **Competition in *H. influenzae*. **B. **Competition in *A. pleuropneumoniae*.

Figure 7 shows the extent to which cells preferentially take up genetically marked conspecific DNA in the presence of competing DNA from their own strain or another species. This is a more sensitive measure of uptake bias than the uptake of pure DNAs tested above. Fig. 7A shows the results of uptake-competition assays using *H. influenzae *cells and a constant amount of *H. influenzae *chromosomal DNA carrying a novobiocin resistance allele. As expected, unmarked *H. influenzae *DNA competed strongly but *B. subtilis *DNA, which does not contain over-represented USS-like repeats, did not [36]. *A. pleuropneumoniae *DNA did not compete for uptake. Fig. 7B shows that *A. pleuropneumoniae *took up its own DNA in preference to both *H. influenzae *DNA and *B. subtilis *DNA. These results confirm that the DNA uptake machineries of both *H. influenzae *and *A. pleuropneumoniae *discriminate strongly in favour of DNAs containing their own USS type. *H. parasuis *DNA was also tested; it did not compete for uptake by *H. influenzae *(Fig. 7A), but competed with *A. pleuropneumoniae *DNA for uptake by *A. pleuropneumoniae *to an extent consistent with the density of *Apl*-type USS in its DNA.

We did not test whether cells could discriminate between DNAs from species in the same subclade. However, as an earlier measure of relatedness among the *Pasteurellaceae*, Albritton *et al*. examined the ability of DNAs from various species to compete with *H. influenzae *DNA for uptake by competent *H. influenzae *cells [37]. The ability to compete for uptake correctly predicted the USS distributions we have found: DNAs from *A. actinomycetemcomitans *and *P. multocida *(*Hin *subclade) competed strongly (54% and 44% as well as *H. influenzae *DNA), but DNA from *A. pleuropneumoniae *(*Apl *subclade) competed only poorly (7%). Although the other sequenced species were not tested, the competition shown by DNA of non-sequenced Pasteurellacean species is likely to predict the USS types they contain. Thus the strong competition Albritton *et al*. observed by *H. parainfluenzae, H. aphrophilus, H. paraphrophilus *and *P. pneumotropica *DNAs suggests that they likely carry *Hin*-type USSs. Consistent with this, *H. parainfluenzae *is known to have *Hin*-type USSs [15], and Christensen *et al*.'s rRNA and protein trees place *P. pneumotropica *close to *P. multocida *with reasonable bootstrap support; their rRNA tree also places *H. aphrophilus *and *H. paraphrophilus *close to *H. influenzae *with modest bootstrap support. In contrast, Albritton *et al*. found that DNAs of *P. ureae, A. lignieresii *and *A. equuli *competed very poorly with *H. influenzae *DNA. These taxa are closely linked to *A. pleuropneumoniae *in Christensen *et al*.'s rRNA tree, and *A. lignieresi *is the sister taxon to *A. pleuropneumoniae *in their protein trees, supporting the hypothesis that they have *Apl*-type USSs [16,17].

The shared features of the Pasteurellacean USS types may reflect generalized features of the DNA uptake process. The 9 bp USS cores may match the size of the recognition domain of the as-yet-unidentified DNA receptor protein, and are similar in length to the 10 bp Neisseria core. The conservation of segment 2 and segment 3 in the Pasteurellaceae is intriguing, as conserved flanking motifs are not seen in *Neisseria*. It may be significant that the spacings between the USS core, segment 2 and segment 3 correspond roughly to single turns of helical DNA. We know it cannot be the case that *H. influenzae *cells initiate uptake by threading a DNA end through a membrane pore, because they efficiently take up covalently closed plasmids [38]. However DNA molecules are too highly charged and too stiff (persistence length about 50 nm or 150 bp) to simply pass sideways through the outer membrane. Together the USS core plus flanking motifs may allow the DNA to be sharply kinked (perhaps by strand separation), presenting a compact cross-section for membrane transit. Detailed understanding of the function of USSs will require more complete experimental studies of binding and incorporation of target DNA sequences.

## Conclusion

The eight Pasteurellacean species we analyzed fall into two robust subclades. The genomes of all these bacteria contain homologues of all the *H. influenzae *genes known to be needed for DNA uptake, some of which have recently been inactivated by mutation. All of these genomes also contain high densities of genetically stable repeats, either the well-characterized *H. influenzae *USS or a related sequence, in each case comprising a 9 bp core and two adjacent AT-rich segments. The distribution of the *Hin*-type and *Apl*-type USSs corresponds to the two Pasteurellacean subclades. Competent members of these subclades discriminate between the two USS types, each preferring to take up DNA containing the USS typical of its own genome.

Taken together, these findings are consistent with the following model of the evolution of competence in the *Pasteurellaceae*: The ancestor of the sequenced *Pasteurellaceae *possessed a complete set of functional competence genes and was naturally competent, taking up DNA by a mechanism very similar to that used by *H. influenzae *today. The ancestral genome contained many USSs; these may or may not have been simpler than the USSs in its descendants, but likely had the common motif ANNNGCGGT in the USS core and included the AT-rich segment 2 and much of segment 3. During the initial diversification of the Pasteurellacean subclades the uptake specificity and USS consensus changed in parallel in one or both lineages. This divergence of genomic USSs may have been effectively complete before the divergence of the sequenced species within each subclade, with USS specificities remaining stable since then, although the existence of *Pasteurellaceae *with other diverged uptake specificities cannot be ruled out. Because the USS consensuses within each subclade are so similar, USS specificity will not enable competent bacteria to distinguish between DNAs derived from different species within their subclade. Because many of these DNAs are otherwise sufficiently diverged that recombination is not only inefficient but toxic [37,39], forces other than exclusion of non-self DNA may be responsible for uptake specificity.

**Figure 8:**
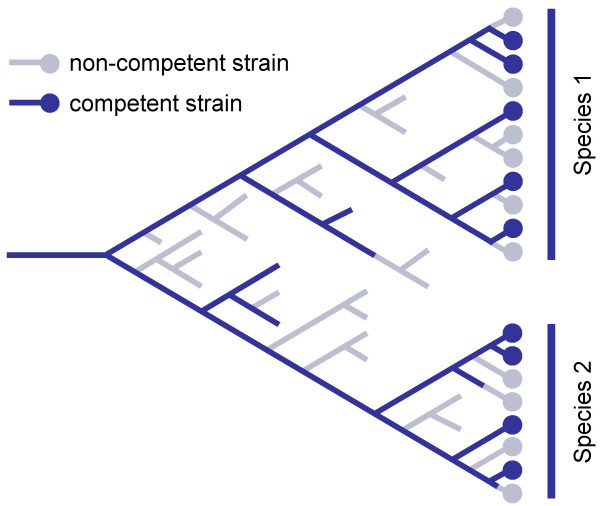
Model for the evolution of competence.

What are the implications for other bacterial families? We suggest that the evolutionary history of competence often follows the pattern shown in Fig. 8. In this model, the ancestors of many bacterial families were naturally competent but competence has been and continues to be frequently lost. Mutations causing loss of competence have not always been strongly selected against, and sometimes may have been actively favoured, so non-competent lineages often persist. However, over the long term the non-competent lineages are selected against, so that all extant bacteria have recent ancestors who were competent. This hypothesis is consistent not only with our family-level analysis but with the extensive evidence of sporadic distribution of competence within individual species [3-6]. The pattern is similar to that seen for the mismatch repair system, where mutants with defects in mutation prevention can experience a short-term advantage but are eventually eliminated by selection against accumulating deleterious mutations [40].

Many questions remain unanswered. How deep is the ancestry of competence? Are some bacterial families ancestrally not competent? Have some modern species completely lost competence? Do genes introduced by conjugation or transduction ever restore competence to non-competent lineages? Thanks to the ever-increasing availability of new genome sequences, answers to these questions will soon be within reach.

## Methods

### Genome and gene sequences

#### Choice of sequences

Complete and annotated genome sequences were available through NCBI for a single isolate each of *P. multocida, M. succiniciproducens *and *H. ducreyi *[30,22,23]. Sequences of four *H. influenzae *isolates were available [41]; we used the fully assembled and annotated sequence of the Rd strain [42]. Sequencing is in progress for two *H. somnus *isolates, we used the more complete 129-PT sequence. Four incomplete *A. pleuropneumoniae *genome sequences were listed at NCBI; however we used our nearly complete sequence of strain L20 (serotype 5 b, sequence available from JN on request). The genome sequences of *M. haemolytica *and *A. actinomycetemcomitans *are also in progress and not yet available at NCBI.

Complete genome sequences with annotations were retrieved from the NCBI website [43] for *H. influenzae *Rd KW20 (NC_000907.gbk), *P. multocida *subsp. *multocida *str. Pm70 (NC_002663.gbk), *M. succiniciproducens *MBEL55E (NC_006300.gbk), *H. ducreyi *35000 HP (NC_002940.gbk), *E. coli *K12 (NC_000913.gbk), *V. cholerae *N16961 (NC_002505.gbk and NC002506.gbk) and *P. aeruginosa *PA01(NC_002516.gbk). Unfinished genome sequences were obtained for *A. actinomycetemcomitans *from [44] and for *H. somnus *129-PT from [45]. Preliminary sequence data for *M. haemolytica *were obtained from the Baylor College of Medicine Human Genome Sequencing Center [46,47]. Open reading frames for genome sequences lacking annotation were identified from the draft sequence using the GLIMMER software package (now available at [48]).

### Phylogenetic analysis

Phylogenetic analysis used the amino acid sequences of the following 12 *H. influenzae *genes and their best homologues in the other 7 Pasteurellaceaen genomes and in *E. coli: gapdH *(HI0001), *lepA *(HI0016), *ffh *(HI0106), *serS *(HI0110), *secD *(HI0240), *dapA *(HI0255), *ruvB *(HI0312), *xerC *(HI0676),*ispB *(HI0881), *secA *(HI0909), *crp *(HI0957), and *dnaJ *(HI1238). Homologues were identified using the BLASTP program (E-val < 10^-50^) in the BLAST package [49]. For each gene, the amino acid sequences from the 9 genomes were aligned using CLUSTALW with output in PHYLIP format [50]. The aligned sequences were inspected, the ends were trimmed to remove sequence missing in any of the 9 genomes, and the alignments of the 12 genes were concatenated to produce a single long alignment.

Phylogenetic analysis of the concatenated alignment used the PHYLIP software package [51]. ProML analysis using maximum likelihood with the JTT method and a gamma-plus-invariant-sites distribution of rates across sites yielded a predicted tree with estimated phylogenetic distances. SeqBoot was then used to produce 100 datasets by bootstrapping resampling; these were put into ProML to generate phylogenetic trees. The final bootstrap analysis was done using the program Consense and the bootstrap values were added to the tree generated with the complete sequence above.

### Homology of USS-encoded peptides

BLAST searches were used to identify those USS-containing *H. influenzae *genes that had homologues in all of *E. coli, V. cholerae *and *P. aeruginosa*. ClustalW was used to align the homologous protein sequences, with the default penalties of 10 for gap opening and 0.2 for gap extension. Analysis was restricted to 43aa segments centred on amino acids encoded by the USS core that showed >50% amino acid identity across all homologues. All gaps within these alignments were tabulated.

### Repeat analysis

The Perl program *repeat_finder *was developed to search genome sequences for abundant short DNA sequences (code available at [52]). It was used to tabulate the occurrences of the 20 most abundant 6-, 7-, 8-, 9-, 10-, 11-, and 12-mers for each of the 8 Pasteurellacaean genomes, along with the number of each expected for a random-sequence genome of that size and nucleotide composition.

All occurrences of the 9 bp putative USS core for each species were identified, and 50-bp sequence segments containing the core plus 11 bases upstream and 30 bases downstream were aligned. The program WebLogo [7,53]) was used to visualize the consensus for each USS. Similar analyses were done for each genome using all singly mismatched occurrences of the 9 bp core, and for *A. pleuropneumoniae *using consensus sequences derived from the two flanking regions.

### Bacterial strains and culture conditions

*A. pleuropneumoniae *serotype 15 (strain HS143) and *H. influenzae *Rd (strain KW20) were grown in Brain Heart Infusion broth (Difco) supplemented with the recommended concentrations of NAD and hemin (*H. influenzae *only), and were made competent by transfer of exponentially growing cells to MIV starvation medium as described for *H. influenzae*[54]. Aliquots of competent cells were stored at -80°C and thawed immediately before use.

### DNA labeling

Chromosomal DNAs of *H. influenzae *Rd and *A. pleuropneumoniae *were labeled by nick-translation with alpha-^33^P-dATP to specific activities of 2 × 10^7 ^cpm/μg. Fragments of about 200 bp centered synthetic USSs (USS-Hin: CCCAAAGTGCGGTTAATTTTTTACAGTATTTTTGGGTTCGAAAT; USS-Apl: GGAAACAAGCGGTCAAATTTGCCGAAAATTTTGCAAATTGGTACCT; USS-Ran: TCTTGTTAGAATCTGAGTGTTATTTAAAT) were PCR-amplified from clones in pGEM [55] using primers with *Bgl*II ends. The amplified fragments were cut with *Bgl*II and end-labeled with Klenow polymerase using alpha-^33^P-dATP, to specific activities of 10^6^–10^7 ^cpm/μg.

### DNA uptake

Competent cells of *H. influenzae *strain KW20 and *A. pleuropneumoniae *strain HS143 (1.0 ml; ~1 × 10^9 ^cfu) were incubated with 150 ng of labeled chromosomal DNA or 20 ng of labeled PCR fragment for 15 minutes at 37°C, followed by 5 minutes incubation with DNase I at 1 μg/ml. Cells were then washed three times at room temperature by pelleting and resuspension in 1.0 ml of MIV, and the radioactivity of the pellets was counted.

### Transformation-competition experiments

Competent cells of *H. influenzae *strain KW20 and *A. pleuropneumoniae *strain HS143 (0.2 ml) were incubated for 15 minutes at 37°C with 100 ng of genetically marked conspecific DNA (MAP7 DNA for *H. influenzae *[54] and *sodC*::Kan DNA for *A. pleuropneumoniae *[25]) mixed with 100, 300, or 900 ng of competing DNA (*H. influenzae *KW20, *A. pleuropneumoniae *HS143, *B. subtilis *or *H. parasuis *(strain Nagasaki) DNA). DNaseI was then added at 1.0 μg/ml for a further five minutes and cells were then diluted and plated on supplemented BHI plates containing 2.5 μg.ml novobiocin (*H. influenzae*) or 25 μg/ml kanamycin (*A. pleuropneumoniae*). Data were plotted using the double-reciprocal method of Sisco and Smith [56].

## Authors' contributions

RJR, WAF, JB and JHEN were involved in the conception and design of the study. RJR carried out the DNA-uptake and competition experiments, drafted the manuscript and produced the figures. WAF carried out most of the bioinformatics analyses. JB analyzed the H. parasuis sequences. ADSC identified and analysed the competence genes in each species. JHEN participated in the bioinformatics analyses. All authors helped interpret the results and write the manuscript, and all read and approved the final manuscript.
